# Micro-alkaline environment enables CO_2_ electroreduction to multicarbons

**DOI:** 10.1093/nsr/nwac230

**Published:** 2022-10-25

**Authors:** Li Li, Yong-Fu Sun, Yi Xie

**Affiliations:** Hefei National Research Center for Physical Sciences at Microscale, University of Science and Technology of China, China; Hefei National Research Center for Physical Sciences at Microscale, University of Science and Technology of China, China; Hefei National Research Center for Physical Sciences at Microscale, University of Science and Technology of China, China

## Abstract

Improving the efficiency towards multicarbons of electrocatalytic CO2 reduction is desirable but challenging. In this perspective, researchers reported the design of micro-alkaline environment could benefit this issue.

CO_2_ electroreduction (CO_2_RR) technology is recognized as a promising option toward the carbon-neutral target set in the Paris Agreement, which enables the upgrade of greenhouse CO_2_ to chemical feedstocks. Particularly, multicarbon products (C_2+_) with higher values are more desired species but are difficult to produce [1]. The development of a successful CO_2_-to-multicarbons conversion system requires addressing the favorable reactive environment.

Gas–diffusion–electrode (GDE) configurations are the most popular systems for CO_2_RR (Fig. 1A). Usually, the GDEs are tightly attached with liquid electrolytes to form the reactive environments and different selections will lead to distinct feedbacks [2]. Over the past 5 years, employing high-alkaline (1–10 M KOH) electrolytes has been a dramatically popular strategy to produce multicarbon products in CO_2_RR [3]. However, the high pH of the bulk electrolyte caused the spontaneous consumption of OH^–^ with CO_2_, and led to the severe CO_3_^2–^ formation, which ultimately resulted in lower carbon efficiency (CE, CO_2_-to-products) and more negative energy balance (EE, power-to-products). Therefore, how to balance the contradictory issues is essential for the CO_2_RR technology to become implementable in industry.

**Figure 1.**
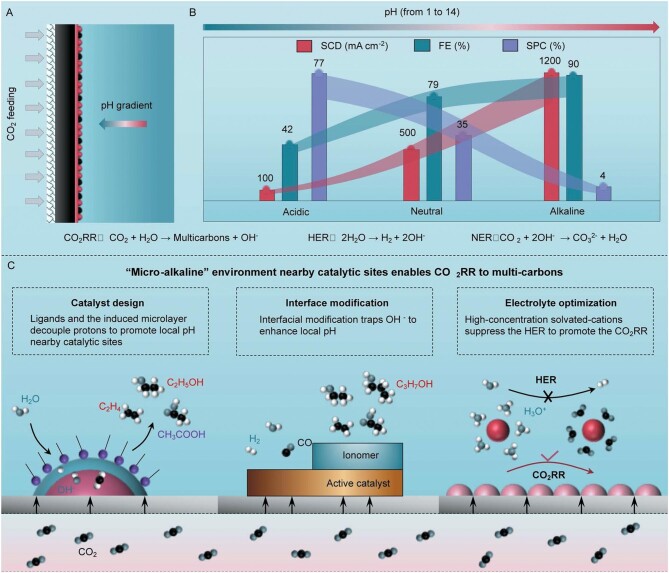
(A) Schematic configuration of GDE and the microenvironment, where the catalysts catalyse CO_2_ to produce multicarbons. (B) The feedback of performances obtained from acidic, neutral and alkaline mediums. (C) Strategies to construct a ‘micro-alkaline’ environment near the catalytic sites and enable CO_2_RR to multicarbons, and the schematic of operations at different scales from the catalyst, interface and bulk electrolyte.

Multiple reactions happen near the interface during CO_2_RR (Fig. 1A). The hydrogen evolution reaction (HER), CO_2_ electroreduction reaction (CO_2_RR) and neutralization reaction between CO_2_ and OH^−^ (NER) all result in the performance indexes of the specific current density (SCD), Faradaic efficiency (FE) and single-path-conversion rate of CO_2_ (SPC), which ultimately determine the energy efficiency and carbon efficiency of a CO_2_RR system. To be specific, in an acidic environment, the HER is kinetically more favorable over the reduction of CO_2_, which leads to high overpotential, poor FE of multicarbons but high CO_2_ utilization. On the contrary, in a high-alkaline environment, CO_2_RR is enhanced due to the suppression of the HER and C–C coupling is greatly promoted to produce multicarbons. However, the high pH of the bulk electrolyte causes the spontaneous NER and leads to negative energy efficiency [4]. More seriously, we should note that the current research on CO_2_RR in high-alkaline electrolytes is actually an energy-wasting process because it would require more energy input to regenerate the used electrolyte than the chemicals produced by itself [5].

The real electrified interface of GDE is a nanoscale component of gas–solid–liquid and the local enhanced pH is beneficial for CO_2_RR to produce multicarbons [6]. Here, we propose the concept of a ‘micro-alkaline’ environment near the cathode surface to meet the favorable conditions for multicarbons production and simultaneously get rid of the troubles of severe CO_3_^2–^ formation by using a high-alkaline electrolyte, by which we hope to encourage more sustainable CO_2_-to-multicarbons conversion technology.

Considering the nano effects and molecular benefits of the pH factor, the strategies at the catalyst level may rely on surface-structuring engineering and functional ligand assembly to reform Cu-based catalysts.

Surface-structuring engineering has proven to be an efficient way to construct local high-pH microenvironments and influence the catalytic reactivity and selectivity of CO_2_RR towards multicarbons. At the same time, highly tunable structures such as the thickness, porosity and orientation can modulate local H_2_O/CO_2_ availability to control reactive pathways and further improve the catalytic performance [7]. More in-depth investigations have suggested that the nanostructures of catalysts not only lower the mass transport of generated OH^−^ to improve the local pH, but also increase the retention time of C_2+_ intermediates within the nanostructures, both of which contribute to the high production of multicarbons. It is worth noting that this strategy always manifests in many forms, such as the thickness, porosity of the catalyst layer and the electrode, which are related to the preparation of the GDE. However, these factors are always neglected or underestimated in reported work. Functional ligand assembly indicates the interactions between the catalyst and the ligand to decouple the H^*^ and create the local ‘micro-alkaline environment’. The locally enhanced electrostatic field decouples protons and promotes local pH. As a result, the Bader charge on the Cu surface is tuned to be chemically favorable for CO_2_ reactant adsorption and C_2+_ intermediate stabilization, thus improving the SCD, selectivity and stability [8].

Besides the design of catalysts, interfacial modification is also an efficient strategy to modulate the local micro-alkaline environment and is potentially more effective than that of catalyst design on a larger scale.

Ionomers and their derivations are the most-used binders for CO_2_RR. Recent work by Bell *et al*. has proven the real role of ionomer coating in optimizing CO_2_RR and accelerating C–C coupling for multicarbons production in neutral electrolytes, which can be described as the formation of an enhanced local pH and CO_2_/H_2_O ratio [9]. In a typical experiment, they coated the Cu surface with different ionomers in sequence and the selectivity towards multicarbons displayed in the order of Naf850/Sus/Cu > Sus/Cu > Cu, which could be ascribed to several factors. First, the different charge between Sustainion and Nafion helped to trap OH^−^ and increase the local pH. Second, the CO_2_ affinity of the ionomer increased the CO_2_/H_2_O ratio near the catalyst surface, thereby enhancing the CO_2_RR. Third, the special sequence of the Naf850/Sus/Cu structure tailored the microenvironment and confined reactants. We may conclude that there are risks that exist in the reported work if researchers only concentrate on the catalysts for CO_2_RR because few of them do not use an ionomer in their catalytic system, while trace amounts of ionomer can make a big difference in the performance. Thus, we suggest paying more attention to the interface modification and the good utilization of this strategy can not only promote the performance towards multicarbons but also lower the unreliability of CO_2_RR systems.

The solvated cations strategy is another feasible method to enable C–C coupling for multicarbons in acidic electrolytes. The inner mechanism is recognized as the molecular-interaction effect, which happens between the solvated cations with the CO_2_ reactant and hydronium ion (H_3_O^+^).

Seminal work has proven that cations are beneficial for selective CO_2_RR with suppressed HER and no CO_2_RR selectivity appeared without cations existing [10]. Further studies suggested that the cations created the enhanced electric field at the catalyst–electrolyte interface, which blocked the H_3_O^+^ and improved the local pH. Consequently, this result augmented CO_2_RR by both blocking the HER process and stabilizing ^*^CO_2_^−^ intermediates. We suggest that efforts in suppressing the HER, enhancing CO_2_ activation and accelerating C–C coupling deserve attention to see the positive profits in CO_2_RR in neutral or acidic electrolytes.

It can be found that many start-up companies on CO_2_ have been established. Therefore, we encourage practical research to balance carbon efficiency and energy efficiency with two principles: (i) balancing the competing process of HER and CO_2_RR to suppress HER or enhance CO_2_RR; (ii) forming a favorable microenvironment for stabilizing CO_2_RR intermediates to accelerate C–C coupling and produce multicarbons. Although neutral electrolytes can balance carbon efficiency and energy efficiency, the problems of CO_3_^2−^ crossover cannot be completely avoided. Current acidic-medium CO_2_ electroreduction shows improved SPC, but the high cell potential and low C_2+_ FE need to be optimized. More advice including the usage of a bipolar membrane (BPM) and tandem electrolyser can be applied to solve the issue. However, due to price factors, these technologies need to be further optimized. We hope these suggestions can encourage more state-of-the-art techniques for creating high-efficiency CO_2_RR in the foreseeable future.
